# Rapid task-dependent tuning of the mouse olfactory bulb

**DOI:** 10.7554/eLife.43558

**Published:** 2019-02-06

**Authors:** Anzhelika Koldaeva, Andreas T Schaefer, Izumi Fukunaga

**Affiliations:** 1 Sensory and Behavioural Neuroscience UnitOkinawa Institute of Science and Technology Graduate UniversityOkinawaJapan; 2 Neurophysiology of Behaviour LaboratoryThe Francis Crick InstituteLondonUnited Kingdom; 3 Department of Neuroscience,Physiology and PharmacologyUniversity College LondonLondonUnited Kingdom; Harvard UniversityUnited States; Vollum InstituteUnited States

**Keywords:** olfactory bulb, dynamic modulation, task switching, Mouse

## Abstract

Adapting neural representation to rapidly changing behavioural demands is a key challenge for the nervous system. Here, we demonstrate that the output of the primary olfactory area of the mouse, the olfactory bulb, is already a target of dynamic and reproducible modulation. The modulation depends on the stimulus tuning of a given neuron, making olfactory responses more discriminable through selective amplification in a demand-specific way.

## Introduction

Behavioural contexts often pose conflicting demands on neural representations of stimuli. A representation that is optimal for one behaviour may be unsuitable for another, as seen in the requirements for discriminating between stimuli vs. generalizing over the same stimuli. Adjusting representations transiently may enable the organism to cope with rapidly changing behavioural contexts.

Previous studies indicate that changes in sensory processing accompany behavioural acquisition of olfactory discrimination as early as in the primary olfactory region of the mouse, the olfactory bulb (OB). Some of these changes depend on the reward association (Doucette and Restrepo, 2008), which in turn also correlate with changes in stimulus sampling patterns (Jordan et al., 2018; Verhagen et al., 2007). In addition, recent reports indicate that the nature of the task, too, is an important determinant. For example, as mice learn olfactory discrimination tasks over days, how the OB output changes during this period depends on the difficulty of the task, where the change in response decorrelation depends on the similarity of odours used (Chu et al., 2016; Yamada et al., 2017). However, some of these changes may be associated with animals becoming familiar with the stimuli over days (Chu et al., 2016). Importantly, whether such task-related changes in the OB are dynamic, and how they are implemented remain unclear.

Here, we investigate how behavioural demands shape olfactory responses of OB output as mice switch between tasks that differ in difficulty. We find that olfactory processing in the OB in response to identical stimuli changes rapidly with task demands, in a manner that is suited to the task at hand.

## Results

**Figure 1.**
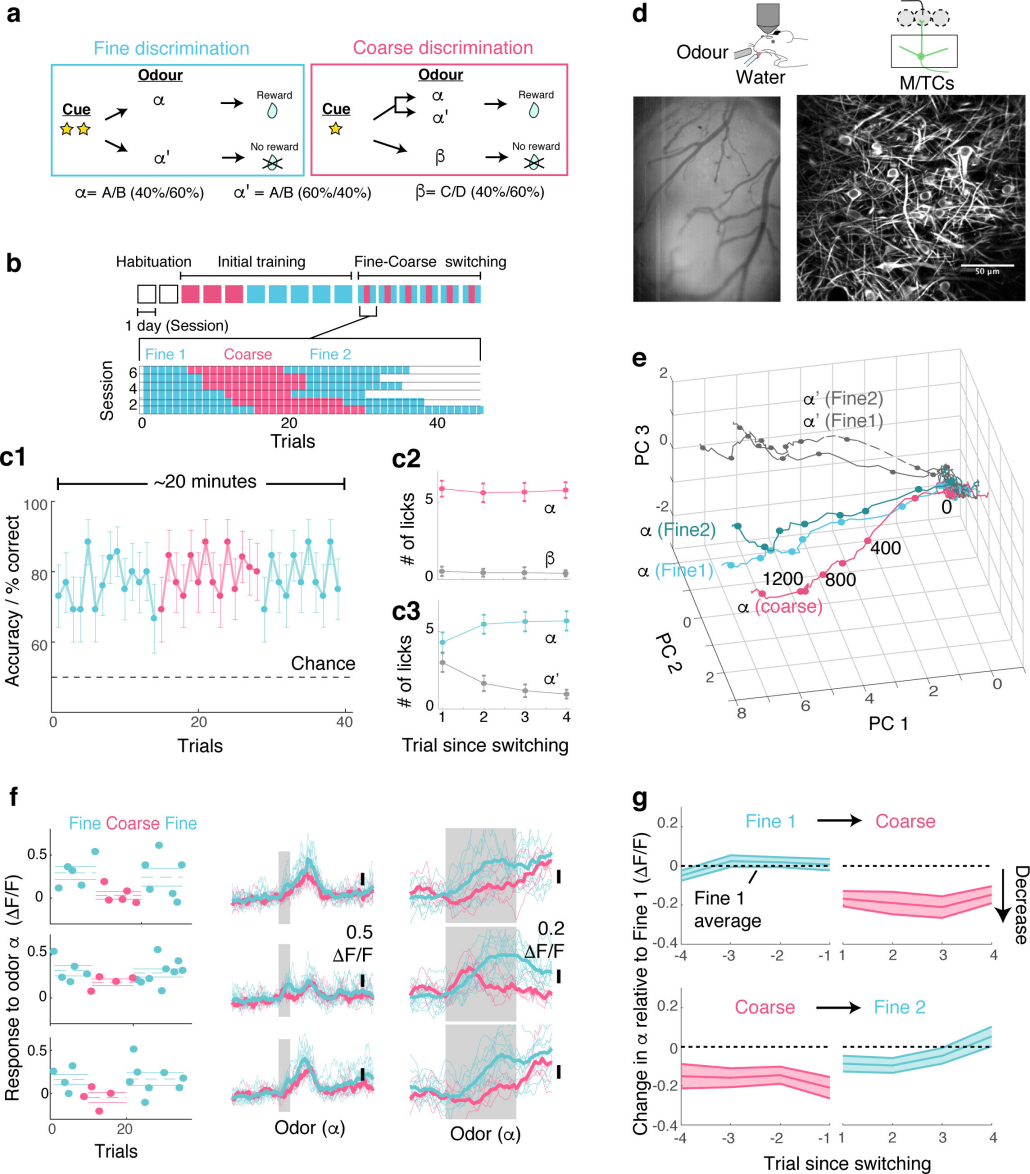
OB output neurons change olfactory representation rapidly and reproducibly with demands. (**a**) Structure of discrimination tasks. (*Left*) Fine discrimination. A trial starts with two flashes of an LED. A rewarded stimulus (α) is a mixture of A and B odours at a concentration ratio of 40:60% (α-odour), associated with a water (reward). In a non-rewarded trial, the A/B mixture is presented at a concentration ratio of 60:40% (α’-odour), and no reward is given. (*Right*) Coarse discrimination. A trial starts with one flash of an LED. The rewarded odour is the same A/B 40/60 mixture as in fine discrimination. A non-rewarded stimulus is a mixture of different odours (β). On some rewarded trials, α’ odour is presented to assess whether mice generalize across both A/B mixtures. (**b**) Timeline of experiment. Each session lasted ~20 min, and occurred once a day. *Inset*: Example of switching sessions used for a typical animal (six sessions shown). Variable epoch length ensures that at least four rewarded trials appear per epoch. (**c**) Trial-by-trial average performance across five mice for all trials (**c1**), as well as trials around switching (**c2,c3**). Mean and s.e.m. are shown. (**d**) Imaging configuration. GCaMP6f fluorescence from mitral and tufted cells was imaged with a two-photon microscope through a chronic window (*middle*) in head-fixed mice performing task switching. *Right*, an example field of view. Scale bar = 50 μm. (**e**) Trajectories corresponding to the α and α’ odours during fine and coarse discrimination, plotted as trajectories in the first three principal components. Pseudo-population response constructed from all ROIs (n = 353, five mice). (**f**) *Left*, Trial-by-trial α odour response (average amplitude during 1 s odour presentation) for example ROIs. Mean and s.e.m. are shown as horizontal lines. *Middle*, Corresponding transients averaged for each task type. *Right*, The same traces as in the middle panel, but zoomed into the odour period. (**g**) Time course of change among significantly modulated ROIs. The response amplitude for an odour relative to response amplitudes during the first fine discrimination epoch (mean ±s.e.m.; n = 42 ROIs, five mice). The observation holds true when the rewarded trials are not preceded by a non-rewarded trial (data not shown).

To study how behavioural demands influence olfactory processing, we used two olfactory discrimination tasks, namely, coarse versus fine discrimination (Figure 1A). Olfactory stimuli used in the coarse discrimination task are easily distinguishable, while stimuli employed in the fine discrimination task are more similar (Figure 1—figure supplement 1). In both tasks, the rewarded odour α (S+ odour) was a mixture of two odorants (A and B), mixed at a concentration ratio of 40%/60%. The nature of the task depended only on the non-rewarded odour (S- odour). In the coarse discrimination task, the S- odour was odour β, comprising odorants C and D, which are not present in the rewarded stimulus. On the other hand, the S- odour used in the fine discrimination task was odour α**’,** made by mixing odorants A and B, but mixed at a different concentration ratio (60%/40%). By using the rewarded odour, α, in both tasks, that is, by making the odour identity and reward association consistent, we isolate the influence of task demands when investigating neuronal responses.

**Figure 1—figure supplement 1.**
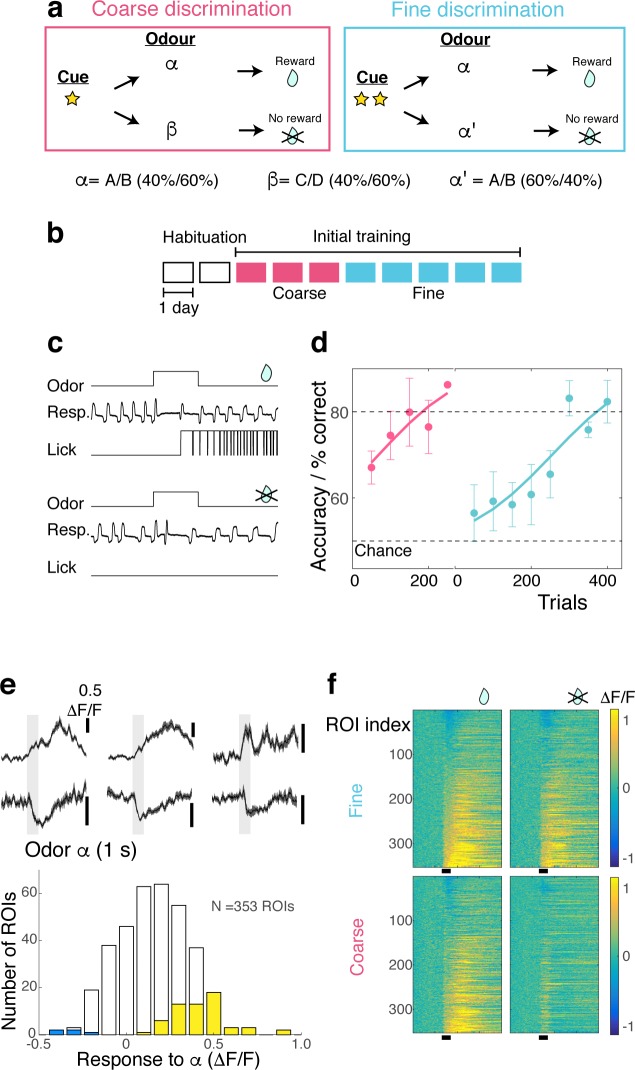
Method for imposing different behavioural task demands. (**A**) Structure of discrimination tasks.* Right*, Fine discrimination. A trial starts with two flashes of an LED. The rewarded stimulus (α) is a mixture of A and B odours at a concentration ratio of 40:60% (α-odour), associated with a water reward. In a non-rewarded trial, the A/B mixture at a concentration ratio of 60:40% (α’-odour) is presented, but no reward is given. *Left*, Coarse discrimination. A trial starts with one flash of an LED. The rewarded odour is the same as in fine discrimination. A non-rewarded stimulus is a mixture of different odours (β). In some rewarded trials, α’ odour is presented to assess whether mice generalize across both A/B mixtures. (**B**) Schematic showing timeline of initial training before mice were subjected to switch training. Each box represents one session, which occurred once a day. Following two days of habituation, mice went through on average three sessions of coarse discrimination training and five sessions of fine discrimination training. (**C**) Examples of correct behavioural responses for rewarded trials (top; correct hit) and for non-rewarded trials (bottom; correct rejection). An odour is presented for 1 s. and a reward is delivered 3 s after onset of the odour. (**D**) Learning rates for head-fixed mice used in the experiment (n = 5 mice) corresponding to initial training days as in (B). Mean and s.e.m. shown. (**E**) *Above*. Examples of excitatory and inhibitory responses evoked by S+ odour (gray bar, 1 s). *Below*. The distribution of responses evoked by S+ odour. Yellow and blue bars = significantly excitatory and inhibitory responses, respectively (see Materials and methods under ‘Odour response’). Scale bar, 0.5 ΔF/F. (**F**) Calcium transients as color maps in response to rewarded (*left*) and non-rewarded (*right*) odours for the tasks indicated. ROIs are sorted by the S+ response amplitude. n = 353 ROIs, five mice.

**Figure 1—figure supplement 2.**
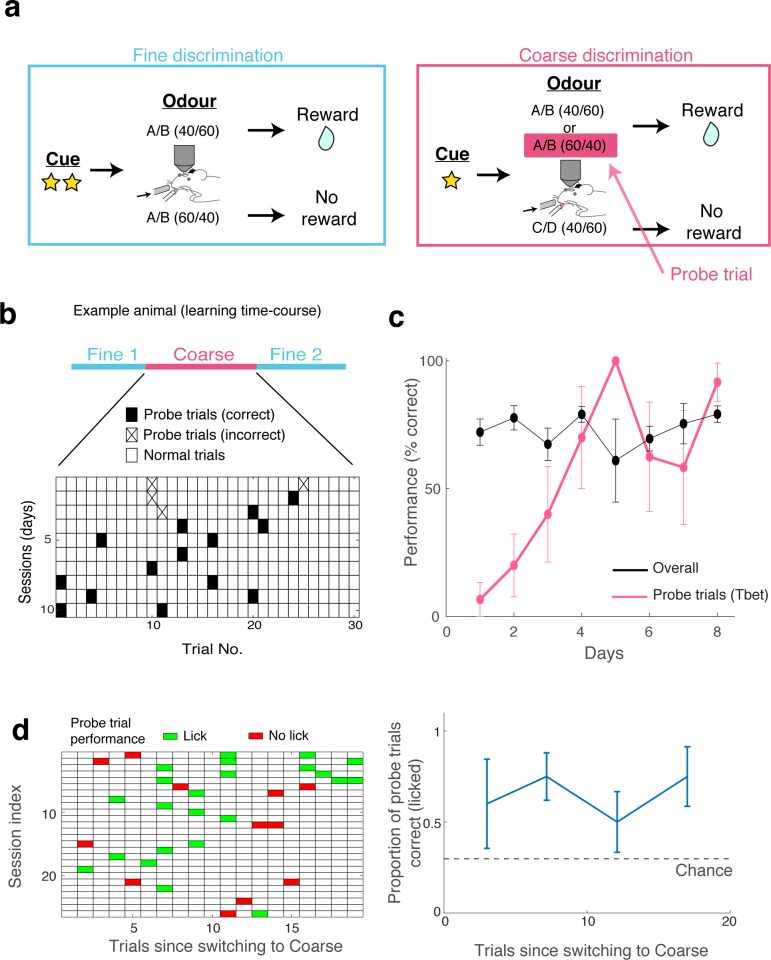
Task-switch learning and probe trials. (**A**) Behavioural paradigm for task switching. Mice are trained to perform fine discrimination blocks before and after a coarse discrimination block within one session. The design of the coarse discrimination is such that mice are forced to generalise closely related odours. On a given rewarded (S+) trial, the odour was either A/B in a 40/60 mixture or A/B in a 60/40 mixture. This latter stimulus appears as the non-rewarded odour in the fine discrimination session. Therefore, the A/B (60/40) trial during the coarse discrimination block serves as a test to see whether mice genuinely perform tasks differently, so they are termed ‘Probe trials.’ (**B**) A typical example showing the performance of a mouse during the probe trials over several days (one session per day). Trials during coarse discrimination are shown as tiles. Probe trials with an incorrect response (i.e., no lick) are marked with a cross, while those with a correct response are shown as filled tiles. (**C**) On average, mice learned to perform task-switching in 4 days, as seen in the performance accuracy in these probe and overall trials. N = 5 mice for Tbet-Cre x Ai95D experiment (average of 1.6 ± 0.4 trials per mouse per session). (**D**) Switch performance for all trials used in the imaging sessions (five mice). Trials are shown from the time of switching from fine discrimination epoch one to coarse discrimination. *Left*: The lick performance for each probe trial is colour-coded, where green indicates the presence of lick (significantly more than during the baseline period, based on Poisson distribution of lick count during the baseline period), and red indicates the absence of lick in response to α’-odour presentation. *Right*: Summary showing the average lick performance on probe trials binned into 4 phases of coarse discrimination epoch. Chance level is the average probability of observing a lick in response to a’-odour during fine discrimination epochs. Mean and s.e.m. shown (average of zeros and ones, with each point reflecting approximately seven trials).

**Figure 1—figure supplement 3.**
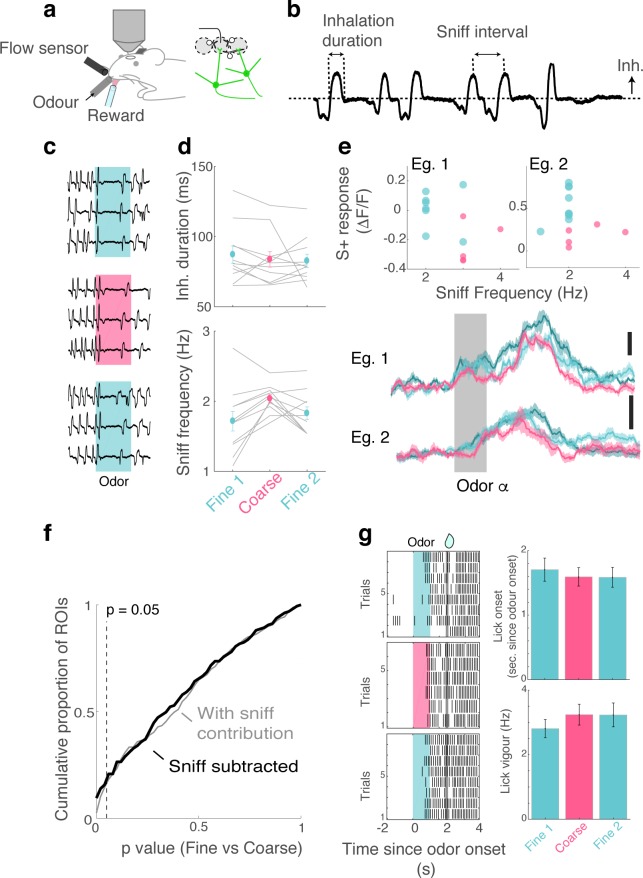
Movement and sniff patterns do not contribute to task-dependent differences. (**A**) Experimental setup. A flow sensor was placed in close proximity to the right nostril to monitor respiratory rhythms while the odour was presented to the left nostril. A lick sensor was a small beam break sensor placed around a water delivery port. (**B**) Explanation of sniff parameters measured. (**C**) Example of respiratory traces around the time of odour presentation. Inhalation is plotted upwards. (**D**) Summary of sniffing patterns showing the average duration of inhalation (*above*) and the number of inhalations taken during the 1 s odour presentation (*below*). (**E**) Examples of dependence on sniff: odour-evoked response amplitudes of two example ROIs against sniff frequency for individual trials during fine discrimination (turquoise) and coarse discrimination (magenta). Average transients for the same ROIs (*below*). (**F**) Distribution of p-values where dependency on sniff frequency is eliminated thorough linear regression (fitted for rewarded odour responses during fine discrimination) in black, and overall p-value distribution where sniff contribution is not subtracted (gray). (**G**) Lick patterns following rewarded odour presentation. *Left*, Examples from one imaging session. Ticks represent occurrence of licks. Each tick represents the onset of licking, relative to the onset of odour presentation. *Right*, Summary showing the timing of the first lick after odour onset (*above*) and frequency of licking for a 3 s window before reward delivery (*below*).

**Figure 1—figure supplement 4.**
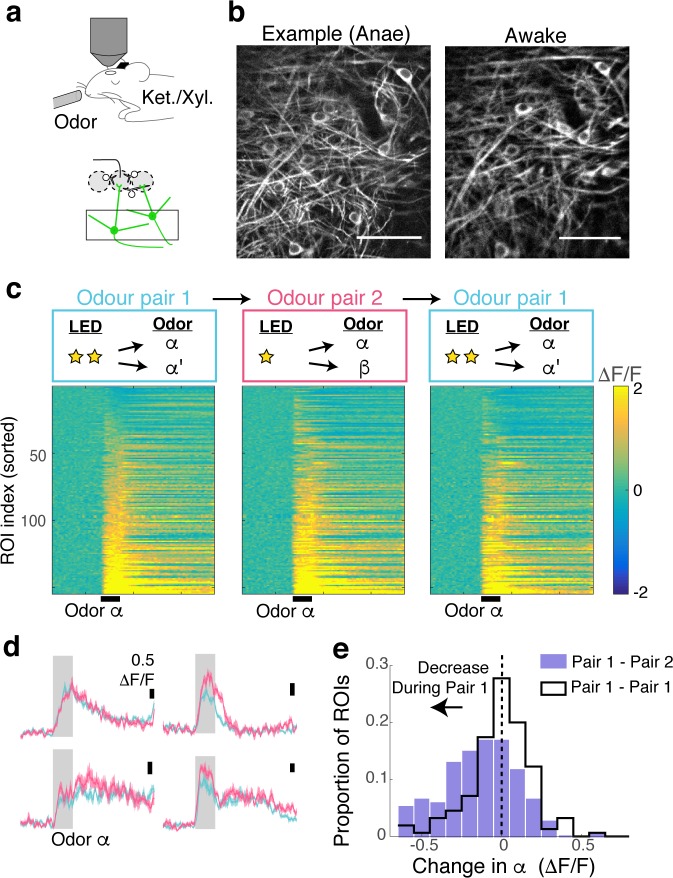
Task-dependent modulation observed during behaviour is absent under anaesthesia. (**A**) After the behavioural experiments, mice were anaesthetized with ketamine and xylazine (i.p.). (**B**) Example fields of view from an example animal show the same location imaged during anaesthesia (left) and during behavioural experiment (right). Scale bar = 50 μm. (**C**) Calcium transients for 155 ROIs imaged, in response to the same rewarded odour. (**D**) Average transients from four example ROIs in response to the rewarded odour during fine (turquoise) and coarse (magenta) stimulus presentations. Scale bar = 0.5 ΔF/F. (**E**) Histogram of task-dependent change in α odour responses (response during fine discrimination – response during coarse discrimination).

Following sequential training for coarse and fine discrimination over two weeks, head-fixed mice were trained to switch rapidly between the two tasks within the same imaging session (Figure 1a,b). A typical session lasted approximately 20–30 min, consisting of two fine discrimination epochs (designated ‘Fine 1’ and ‘Fine 2’) and one coarse discrimination epoch that occurred between the two fine discrimination epochs, with each epoch 15–25 trials long. This design was used to control for time-dependent effects. On some trials during the coarse discrimination, odour **α’** (the 60A/40B mixture) was presented as a rewarded odour (‘probe’ trials). This modification forced mice to generalize over A/B mixtures (**α and α’**) during coarse discrimination, and to discriminate between the **α** and **α’** odours during fine discrimination. Since our focus is to study how the α-odour response changes with task demands, probe trials were presented, on average, 1.6 times per session only. Over the course of approximately four days, mice learn to perform the task switching with high accuracy (Figure 1c, Figure 1—figure supplement 2).

To assess the effect of task demands on OB processing, olfactory responses of the principal neurons, mitral and tufted cells (M/TCs), were imaged in trained mice during behaviour using a two-photon microscope (Figure 1d). A genetically encoded calcium indicator, GCaMP6f (Chen et al., 2013), was expressed conditionally in M/TCs in the OB by crossing the Ai95D mouse line (Madisen et al., 2015) with the Tbet-ires-Cre mouse line (Haddad et al., 2013). Imaging was accomplished through a previously implanted chronic imaging window (Holtmaat et al., 2009).

To visualize if and how M/TC responses as a whole are modulated by tasks, calcium transients from all regions of interest (ROIs; 353 ROIs from five mice) were expressed as pseudo-population vectors and plotted as trajectories in the first three principal components (Figure 1e). Despite the fact that the odour is identical, the trajectory for the α odour during coarse discrimination lies distinctly away from that for fine discrimination. On the other hand, the trajectories for odour **α** from the first and second epochs of fine discrimination superimpose closely. This indicates that olfactory representation in the OB changes with task, but reversibly.

At the level of individual cells, a subset of MTCs was found to change its responses significantly with task (42/353 ROIs; 17/353 ROIs in shuffle control show significant change). Single-trial analysis revealed that lick and sniff patterns do not explain this task-related change (Figure 1—figure supplement 3). In fact, when variability arising from sniff patterns was removed through linear regression, a greater proportion of ROIs were found to be significantly modulated by task (56/353 ROIs; Figure 1—figure supplement 3). In addition, this task-related modulation is absent in mice anaesthetized with ketamine and xylazine (Figure 1—figure supplement 4). Importantly, the change is present immediately after task switching (Figure 1f,g). Among the task modulated ROIs, significant change is observed even in the first trial after switching from fine to coarse discrimination (mean change in the α odour response during 1^st^ coarse trial = −0.17 ± 0.04 ΔF/F; p<0.01, t-test for equal means, t-score = −4.3, n = 43 ROIs, five mice). Switching back to fine discrimination, responses become comparable to the original amplitudes by the 3^rd^ trial (mean difference in α odour response relative to Fine 1 = −0.05 ± 0.04 ΔF/F; p=0.26, t-score = −1.2), closely mirroring the recovery time course of behavioural accuracy (Figure 1c).

**Figure 2.**
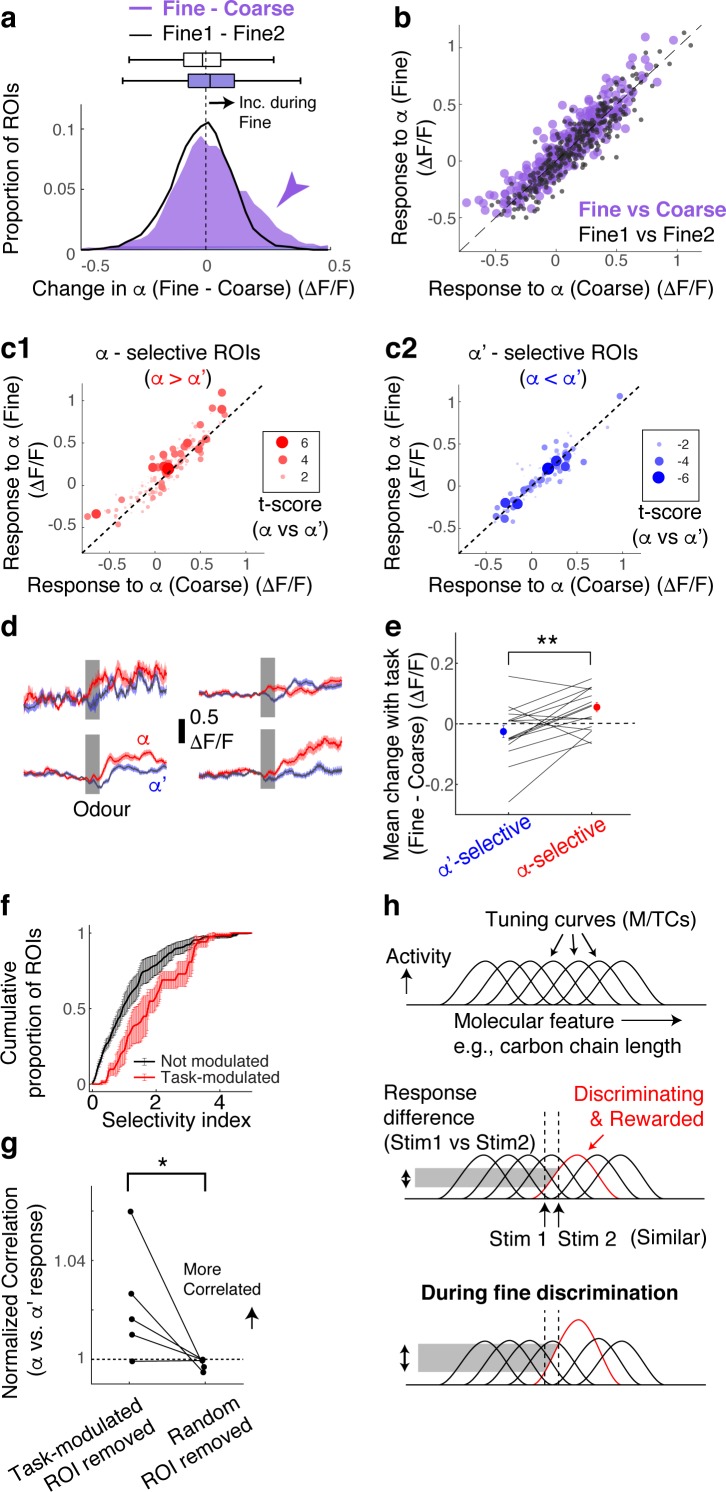
Selective amplification leads to demand-specific enhancement of stimulus decorrelation. (**a**) Histogram of task-dependent change in α responses (response during fine discrimination – response during coarse discrimination; purple). Within-task variability (black line) is shown for comparison. (**b**) Comparison of odour responses during coarse (x-axis) and fine (y-axis) discrimination for all ROIs (purple). Within-task comparison (Fine1 vs Fine2; black points) shown for reference. (**c**) Task-dependent odour response comparison as in (b), but separated by odour selectivity. α-selective ROIs (red) are ROIs with t-scores greater than zero, where the strength of the selectivity is indicated by the size of the marker. α‘- selective ROIs are those with t-scores <0. (**d**) Average transients in response to odour α (red) and odour α’ (blue) from 4 ROIs selective for odour α. Error bar = s.e.m. (**e**) Summary of task-dependent change for odour α for ROIs grouped by selectivity. Each data point = average for each imaged location. N = 21 sessions, five mice. Sessions where probe trials were omitted are excluded from this analysis. (**f**) Distribution of selectivity indices for task-modulated ROIs (red; n = 32 ROIs, five mice) and ROIs without task modulation (black; n = 255 ROIs, five mice). The selectivity index is the absolute value of the t-score for α-responses vs α’-responses imaged during fine discrimination. (**g**) Correlation coefficients between α vs α’ responses, where a subset of ROIs is removed and normalized to when a full set of ROIs used. Each coefficient is from ROIs from individual animals, instead of imaging location, due to a small number of ROIs in some imaged locations (n = 5 mice). (**h**) Schematic of finding. MTCs tuned to different ‘molecular features,’ for example, distinct carbon chain lengths, have different tuning curves along this axis. Some MTCs respond differently to two similar stimuli (stim 1 and stim 2), which contribute the most to discrimination. MTCs that are selectively tuned to the rewarded odour are enhanced when mice need to discriminate between the two similar odours, resulting in enhanced discriminability.

Overall, M/TCs tend to increase their responses during fine discrimination (Figure 2a,b). The increase is particularly pronounced when mice show clear evidence of switching, as assessed by the performance during probe trials (Figure 2—figure supplement 1).

**Figure 2—figure supplement 1.**
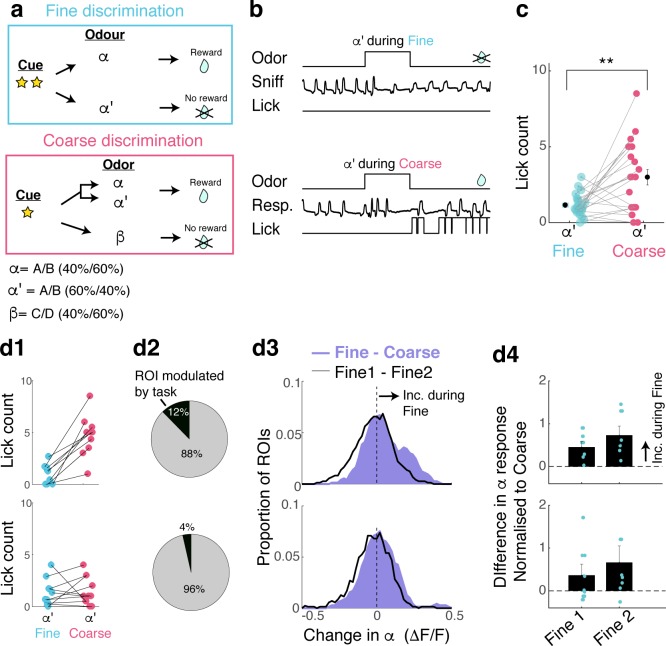
Task-related change is prevalent in sessions with clear evidence of switching. (**A**) Task structures as before. (**B**) Example of trials where a 60A/40B mixture (α’ odour) was presented as a non-rewarded odour during fine discrimination (above) and as a rewarded odour during coarse discrimination (below). (**C**) Summary comparison of lick response for the same odour but during different tasks. (**D**) Analysis of data from sessions in which mice switched vs those in which they did not. All data are from well-trained animals, with imaging taking place on 3–6 days since the beginning of switch training. (**D1**) Definition of switching based on licking pattern following α‘ odour presentation. Above: sessions in which mice switched, defined by a significant increase in licking during α‘ trials during coarse discrimination. N = 10 imaging sessions, five mice. Below, Definition of sessions in which mice did not switch, where licking patterns during α ‘odour trials do not differ during fine and coarse discrimination. N = 11 imaging sessions, five mice. (**D2**) Proportion of ROIs that are modulated by task for sessions with evidence of behavioural switching (above) and sessions with no such evidence (below). N = 146 ROIs and 141 ROIs, respectively. (**D3**) Histogram of change in α odour response amplitudes, across task (Fine - Coarse discrimination; purple) and within task (Fine1 – Fine2, black line). N = same as in D2. (**D4**) Task-dependent change in α response amplitudes; summary representing individual imaging sessions (N = 10 for sessions with behavioural switching, n = 11 for sessions with no behavioural switching).

**Figure 2—figure supplement 2.**
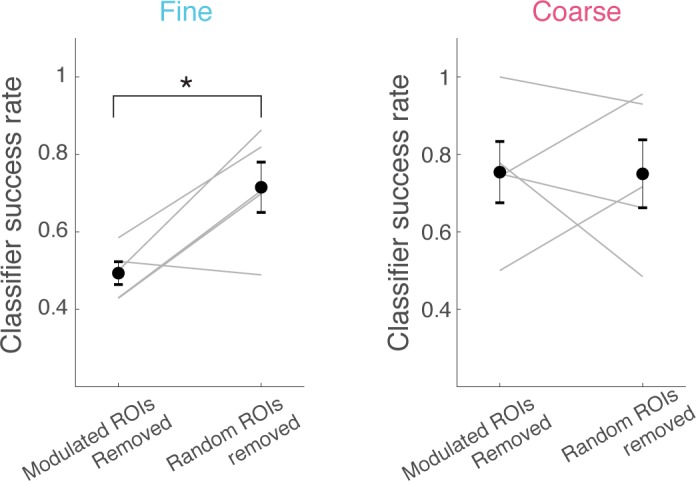
Role of task-modulated neurons in task-dependent stimulus discrimination. Linear classification performance for discrimination of α vs α’ odours (trials from fine discrimination epochs), where a subset of ROIs is removed. Each success rate plotted is from an individual animal, as in Figure 2g.

**Figure 2—figure supplement 3.**
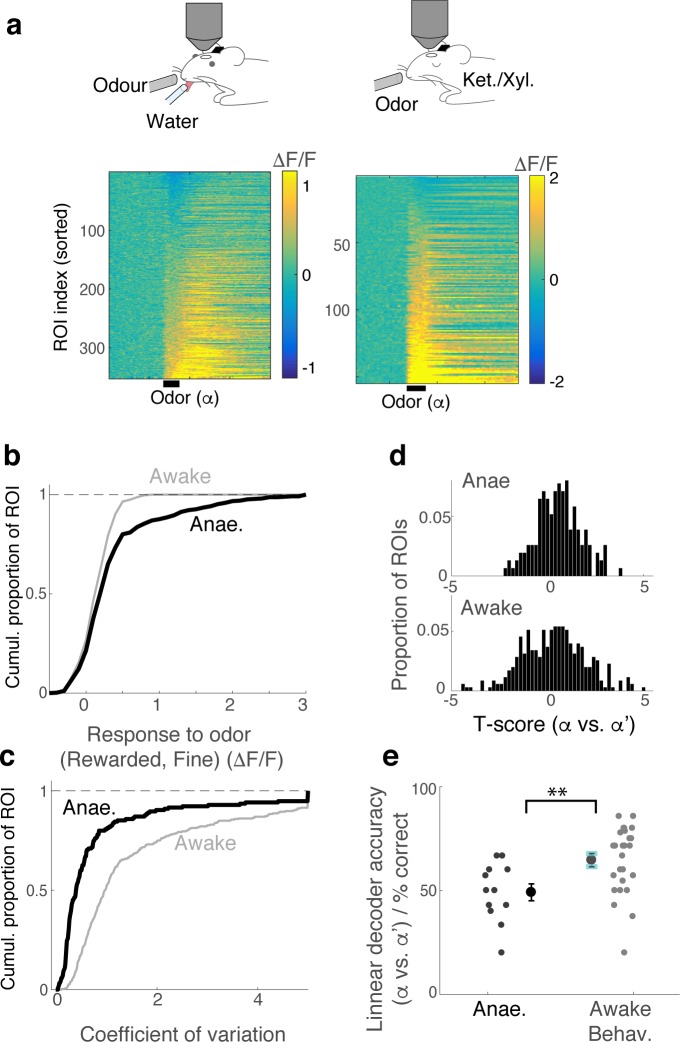
Responses are more reliably discriminated in behaving mice. (**A**) GCaMP6f fluorescence change is represented in a color map for 353 ROIs imaged from awake, behaving mice (26 imaging sessions, five mice). Left; Responses are from mice performing the fine discrimination task. Right; Following behavioural experiments, mice were anaesthetized with ketamine/zylazine and M/TCs imaged (12 imaging sessions, three mice). Responses are from block 1, where odour α and odour α’ were presented closely in time. (**B,C**) Comparison of cumulative histogram of response amplitudes (**B**) and coefficient of variation (**C**) for odour α (average during 1 s after valve opening). (**D**) Comparison of t-score distribution (α odour response vs α’ odour response amplitudes) for ROIs imaged from anaesthetized (above) and awake, behaving (below) mice. (**E**) For each imaged location, a linear classifier was trained on a subset of α- and α’- trials. Its performance for test trials is shown as % correct. (average accuracy = 49.1 ± 4% correct for anaesthetised vs. 64.6 ± 3.0% correct for awake, behaving case; p=0.006, Wilcoxon rank sum test; n = 12 imaged locations, three mice for anaesthetized mice vs. 26 locations, four mice for awake).

**Figure 2—figure supplement 4.**
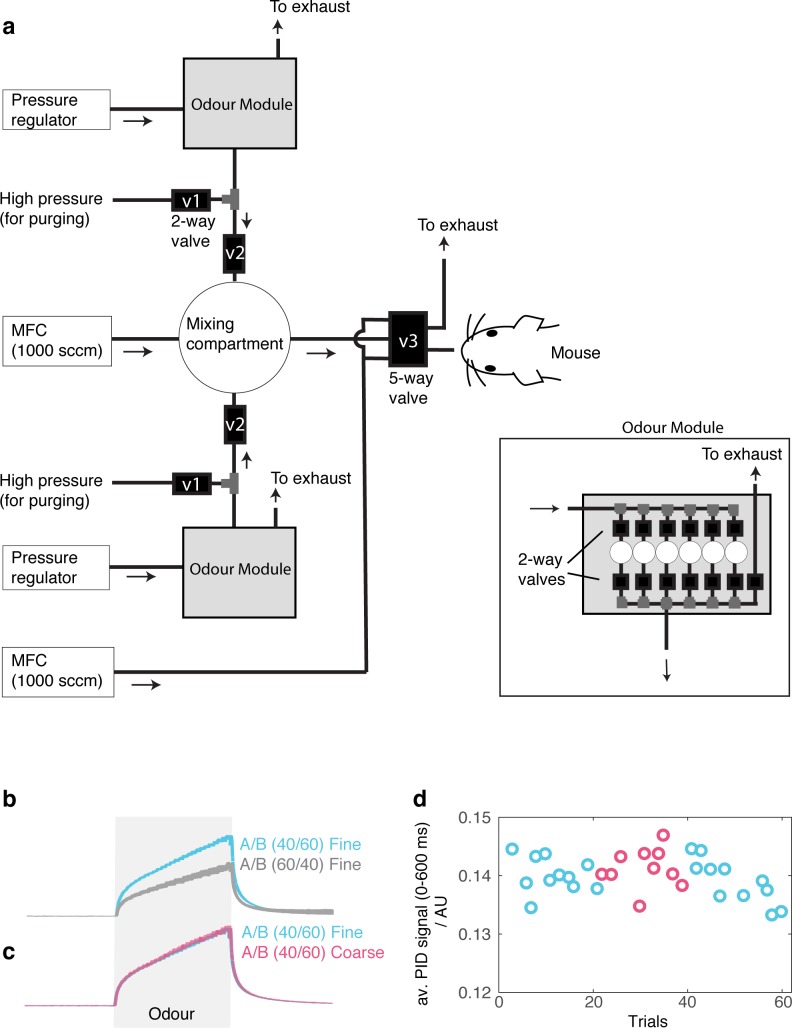
PID signals show stable odour pulses. (**A**) Olfactometer design for mixing two odorant streams at desired concentrations for generating binary mixtures. For each odorant, filtered and regulated air is passed through a selected odour canister by opening a pair of two-way solenoid valves ('Odour module'). Odourized air is passed through a second, fast solenoid valve (‘v2’) in a pulsatile manner. Pulses of odourized air are mixed in an enlarged compartment (a 50 ml flask) to remove transients. The odour concentration can be set by controlling background air flow and odorized pulse parameters (duration and frequency of rectangular pulses). Mixed air passes through thin PTFE tubing (O.D., 3.2 mm) and is presented to the animal when the 5-way solenoid valve is actuated. (**B**) For characterizing the stimulus profile, a photo-ionization detector was placed in front of the odour port, in place of the mouse. Instead of the usual binary mixtures, the test used a mixture of 1 odorised stream and one blank stream (passing air through an empty odour vial) mixed in the same way to test the concentration of each component separately. PID signals from trials where the composition of the tested odour is 60% (cyan) vs 40% (gray), corresponding to the composition of an odour ‘A’ in S+ and S- trials, respectively. (**C**) Odour concentrations (PID signal) measured during coarse and fine discrimination for the switching paradigm are overlaid. Mean +- standard error of the mean shown (~15 trials each). (**D**) Time-course of odour concentrations to test the depletion level during a task-switch session. The inter-trial interval was approximately 20 s. Data shown are the average PID signal during the first 600 ms of odour period.

**Figure 2—figure supplement 5.**
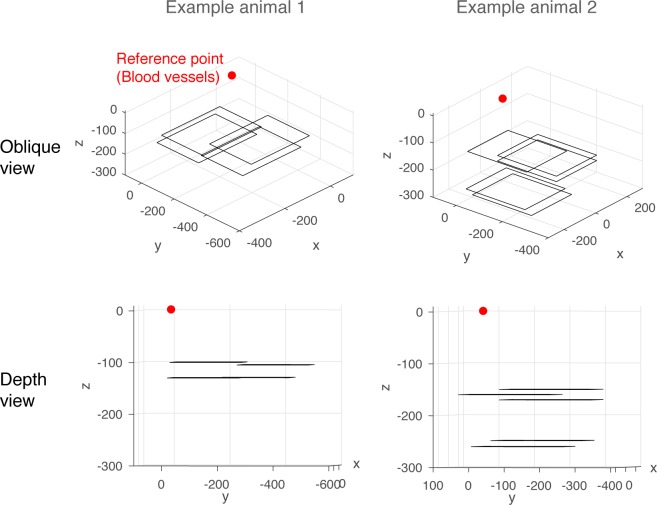
Examples of imaged planes. For each imaging session, images were obtained from a 256 μm x 256 μm field of view from a single plane. In each animal, imaging sessions took place over multiple sessions, depending on the quality of the optical window, as well as the presence of odour-responsive neurons. The panels show all imaged planes from two example mice, with two viewing angles.

**Figure 2—figure supplement 6.**
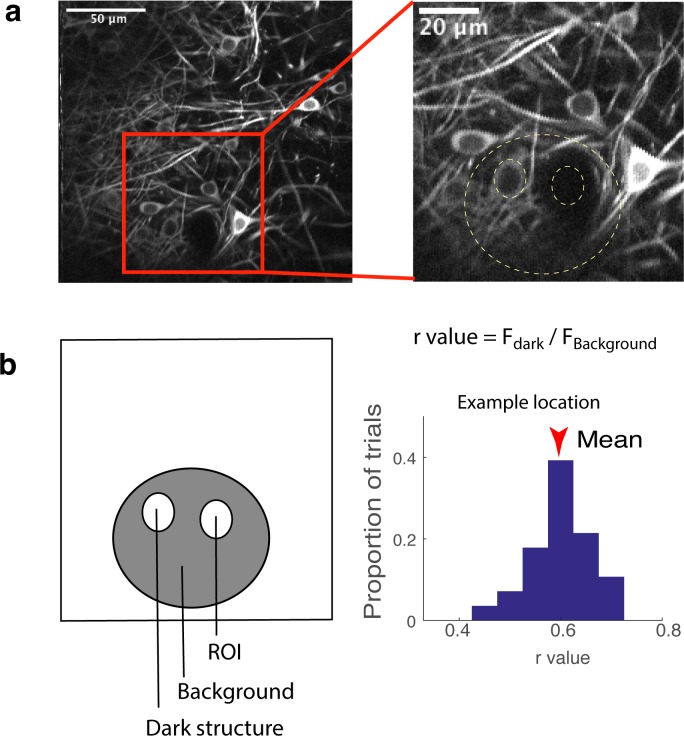
Estimation of contribution from out-of-focus fluorescence. The method is based on Kerlin et al. Briefly, to estimate how much out-of-focus fluorescence contributes to fluorescence within an ROI, the fluorescence of a neighbouring dark object (F_dark_; blood vessel or unstained structure) and the fluorescence of the area surrounding the two objects (ROI and the dark object) are compared. The ratio F_dark_/F_background_ is the estimated fractional contribution from out-of-focus fluorescence.

According to a previous, longitudinal study, when mice learn to perform fine discrimination, there is an accompanying increase in the fraction of divergent responses among M/TCs (Chu et al., 2016). Accordingly, we assessed whether stimulus selectivity is an important parameter in the dynamic, task-related change observed here. To this end, for each ROI, we measured its selectivity to α vs α’ odours using the t-score (or t-statistic), which compares mean response amplitudes (see Methods). Intriguingly, we found that it is the α -selective M/TCs that enhance their responses during fine discrimination (Figure 2c–e; mean change = 0.06 ± 0.02 ΔF/F for alpha selective ROIs; p = 0.008, paired t-test for equal means between α-selective and α’-selective ROIs; n = 21 sessions, five mice).

As a result, stimulus-selective neurons are over-represented among task-modulated M/TCs (Figure 2f; p = 0.01, 2-sample K-S test for equal distributions, K-S statistic = 0.33, n = 22 task-modulated ROIs and 201 non-modulated ROIs from sessions with probe trials). Notably, 24% of stimulus-selective neurons were significantly task modulated, compared to 10% of the entire population of neurons, together suggesting a critical role that modulation plays in discriminating similar stimuli. Consistently, when these task-modulated ROIs are removed from the analysis, the population of M/TC responses to α and α’ odours became more correlated (Figure 2g; mean % change in Pearson’s Correlation = 2 ± 1 when modulated ROIs were removed, and 0.2 ± 0.1 when random ROIs were removed; p=0.04, paired t-test, n = 5 mice) and the decoder performance deteriorated specifically for fine discrimination (Figure 2—figure supplement 2). We note that a general increase in response amplitudes alone does not explain our result, as an overall increase in response magnitude in an anaesthetized preparation does not lead to enhanced stimulus selectivity and discriminability (Figure 2—figure supplement 3).

## Discussion

Overall, we find that rapid, task-dependent modulation of odour responses in the primary olfactory area occurs dynamically to enhance odour representation to suit the behaviour. We find that modulation occurs even when the stimulus-reward association is identical, isolating context as the only variable. Concomitantly, discriminability of stimulus representations is altered through selective boosting of responses of discriminating neurons (Figure 2h). These divergent responses occurred in about 12% of the neurons we observed, closely matching a previous report (Chu et al., 2016).

Similarly, task-related modulation is not easily explained by the difference in the stimulus statistics (frequency of A/B mixture presentations), as the modulation is absent in mice anaesthetized with ketamine and xylazine (Figure 1—figure supplement 4), although we cannot exclude that the influence of stimulus-statistic might be state-dependent. How, then, might dynamic and selective modulation arise in the OB? Unusually for a primary sensory area, the OB receives dense feedback projections from olfactory cortices (Boyd et al., 2012; Davis and Macrides, 1981; Markopoulos et al., 2012; Otazu et al., 2015; Rothermel and Wachowiak, 2014) as well as neuromodulatory centres (Macrides et al., 1981; McLean and Shipley, 1987; Steinfeld et al., 2015). Among these, possible pathways for selective amplification include direct excitatory feedback, such as a direct excitatory drive from centrifugal inputs onto M/TCs, as described for the AON (Markopoulos et al., 2012), or modulation of inhibitory neurons via some competitive mechanism (Koulakov and Rinberg, 2011), although we cannot rule out a role for neuromodulators. So far, there is no evidence that olfactory signals carried by the cortical feedback are spatially matched to local (glomerular) olfactory representations (Boyd et al., 2015). Whether cortical feedback to the OB indeed forms a pattern akin to an ‘attention-field’ to enhance relevant signals (Reynolds and Heeger, 2009) will be a key topic of future investigation.

Due to the limitations from kinetics and sensitivity of the genetically encoded calcium indicators (Chen et al., 2013), we chose to analyse the 1 s window during odour presentations. This calcium response could therefore potentially reflect a variety of parameters related to behaviour, in addition to a purely sensory component. Indeed, previous experiments demonstrated that mice take less than 1 s to arrive at decisions in similar tasks (Abraham et al., 2004; Rinberg et al., 2006; Uchida and Mainen, 2003). It is therefore not inconceivable that some of the task-dependent differences might reflect aspects of decision variables.

According to models of attention developed especially in the primate visual system, the extent of modulation depends on many factors, including the similarity of the attended feature and the neurons’ tuning, the exact stimuli used, as well as the stages of sensory processing along a hierarchy (reviewed in Reynolds and Heeger, 2009). Indeed, a variety of modulation sizes have been observed at different levels of visual processing: 8% in V1, 26% in V4 (McAdams and Maunsell, 1999; Moran and Desimone, 1985; Motter, 1993), 19–60% in MT and 40% in MST cells (Treue and Maunsell, 1996; Treue and Martínez Trujillo, 1999). In discriminating similar stimuli, neurons that contribute the most are those that respond differently to the stimuli used. Modelling studies suggest that these are most often neurons with a preferred stimulus feature that is shifted slightly away from the stimulus features to be discriminated (Jazayeri and Movshon, 2006). These, in our case, comprise about 12% of the imaged neurons, which closely match previous reports (Chu et al., 2016). Our results suggest that such neurons may be the target of selective and dynamic modulation to enable enhanced discriminability as needed. The ability to induce different task demands and observe the resultant modulation in a primary sensory area may prove useful to understand how the brain implements solutions that meet ever changing behavioural demands.

## Materials and methods

### Animals/surgery

All animal experiments were approved by the institutional veterinarian and ethics committee of OIST Graduate University (2016–3) and have been performed in strict accordance. All recovery surgery was carried out using standard aseptic technique under isoflurane anaesthesia and carprofen analgesia. Adult male mice (6–8 weeks old) from the cross between the Tbet-Cre line (Haddad et al., 2013) and the Rosa26-GCaMP6f line (Madisen et al., 2010) were used. For chronic optical access, a method similar to that of Holtmaat et al. (2009) was used. Briefly, a small, round piece of glass (~1 mm diameter) was cut from a cover slip (borosilicate glass 1.0 thickness) using a diamond knife (Sigma-Aldrich). It was fitted over a craniotomy made above the left olfactory bulb and sealed with cyanoacrylate (Histoacryl, TissueSeal, USA). A custom-made head-plate was attached to the exposed, dried occipital bone using gel superglue and dental cement. Non-steroidal anti-inflammatory treatments (Carprofen, i.p.) were given for 3 days post-operatively. All behavioural sessions began more than 2 weeks after the surgery.

### Olfactometry

Odours were presented using a custom-made flow-dilution olfactometer similar to an earlier design (Fukunaga et al., 2012), except in the control of odour concentrations (Figure 2—figure supplement 4). Odour concentration was set using precise, pulsatile packets of saturated odour into a 50 mL flask, where it was mixed with a steady flow of background air (one slpm) in order to eliminate fast transients in odour concentration (time constant =~ 400 ms). The final odour concentration presented to the animal was approximately 0.5–1% of the saturation level. A binary mixture was generated by mixing two streams of odorized air in the same mixing compartment, and concentrations were verified using a photoionization detector for each odour component (where the tested odour was mixed with a blank control that did not give signals; Figure 2—figure supplement 4). Each odour canister had a large headspace (~40 mL) over the odorants to minimise run-down over time. Teflon tubing and air purge (~5 slpm for 10 s) during the inter-trial interval were used to minimize contamination.

### Go-No-Go olfactory discrimination

Water access in the home cage was restricted, and head-fixed mice went through habituation sessions before discrimination training commenced. During habituation sessions, animals were head-fixed and were presented water from the port. This stage of training lasted 15–20 min a day, until animals were comfortable enough to drink water from the water port. This was typically achieved within two days. Animals were then trained to associate one olfactory stimulus with a water reward (S+ odour) and another olfactory stimulus with no reward (S- odour). The correct response for the S+ trials was to lick the water port for a reward during 2 s after the onset of odour presentation, while the correct response for S- trials was to refrain from licking. The reward was a single drop of water approximately 20 uL in volume. The water port served also as a lick sensor (beam break; PM-F25, Panasonic, Japan), which was coated with black silicone externally to prevent light leakage. Respiratory rhythms were monitored continuously through the contralateral naris using a fast mass flow sensor (FBAM200DU, Sensortechnics, Germany). Odours used were ethyl butyrate and eugenol in the rewarded odour mixture, and methyl salicylate and methyl tiglate in the non-rewarded odour mixture used in the coarse discrimination. The sequence of S+ and S- trials was chosen so that no more than three consecutive trials were of the same type, but otherwise random. On each trial, odour was presented for 1 s, and inter-trial interval was 20 s. Animals were monitored closely and at no point did animal's weight decrease below 80% of the initial weight.

### Imaging

Data in this manuscript were obtained from awake, head-fixed mice engaged in the tasks, as well as anesthetized mice, as indicated, through previously implanted optical windows. Imaging in awake animals took place after approximately 4 days of task-switch training, when mice had acquired the task-switching. In these trained mice, imaging sessions took place for up to 6 days depending on the quality of the imaging window. Each session lasted, on average, for 30 min. The exact number of trials mice performed each day varied depending on the level of motivation. However, to balance time-dependent effects, including bleaching, the same number of trials from the first and second fine discrimination epochs were analysed. That is, the later trials during the 2^nd^ epoch of fine discrimination was truncated from analyses. Imaging in anaesthetized mice took place after the switching sessions. Two-photon fluorescence of GCaMP6f was measured with a custom-fitted microscope (INSS, UK) fitted with a 25x Objective (Nikon N25X-APO-MP1300, 1.1 N.A.), and high-power laser (930 nm; Insight DeepSee, MaiTai HP, Spectra-Physics, USA) at depths 50–400 μm below the surface of the olfactory bulb. Each field view was 256 μm x 256 μm (512 × 512 pixels). Images from a single plane were obtained at ~30 Hz with a resonant scanner. Each day, the stage co-ordinate was zeroed at a reference location, guided by the surface blood vessel pattern using an epifluorescence camera. Imaging location was then chosen as an area not overlapping with previous sessions (based on the x,y and z co-ordinates and field view, and confirmed post-hoc by eye; see Figure 2; Figure 2—figure supplement 5). Modulated cells were found at all depth of imaging, suggesting that both MCs and TCs are subject to task-dependent modulation.

### Analysis

Images were analysed using custom-written routines in Spike2 (Cambridge Electronic Design, UK), Matlab (Mathworks, USA) and macros in Fiji (ImageJ), run on a PC (32 GB RAM, 10 × 2.2 GHz Intel Xeon processors).

#### ROI detection

From an average of ~1000 frames for each imaging plane, somata were manually delineated using an ROI manager (ImageJ). Selected oval regions were confined within the perimeter of the soma, and somata with filled nuclei were excluded. Based on the method by Kerlin et al. (2010) (Figure 2—figure supplement 6), the contribution from out-of-focus fluorescence was approximately 60%.

#### Odour response amplitude

Unless otherwise stated, the average odour response was obtained from ΔF/F values during the first 1 s of odour presentation (after the onset of valve opening). Results are qualitatively similar when measurements are from shorter time windows, but noisier, especially for single-trial analyses.

#### Similarity of S+ vs S- representations

For each pair of responses per imaging location, the relative fluorescence change (ΔF/F values) for all ROIs in the field of view was treated as vectors and Pearson correlation coefficient was calculated.

#### Odour response

Significant responses were defined as those in which the magnitude of odour-evoked fluorescence change exceeded 3x the standard deviation of baseline fluctuations.

#### Task-dependent change

To determine if a task-dependent change was statistically significant, evoked response amplitudes for S+ trials for fine and coarse discriminations were tested for the same distributions (two-sample t-test for no difference, two tailed).

#### Principal component analysis

The method is based on Niessing and Friedrich (2010). Briefly, for each ROI, GCaMP6f transients (ΔF/F values) from each condition (odour and task) were concatenated and principal components obtained using the Matlab function *pca*. Original data were projected on the new components (first three principal components) to obtain trajectories.

#### Stimulus selectivity (T-score)

For each ROI, odour response amplitudes were obtained from α trials and α’ trials. These were compared using the Matlab function for the two means, namely, the two-sampled t-test (ttest2) to obtain a t-score (t-statistic). The T-score was used instead of the commonly used z-score because of the small number of trials.

#### Stimulus selectivity

Selectivity index was defined as the magnitude (absolute value) of the t-score.

#### ROI removal and resultant change in stimulus correlation

For each animal, average odour response from ROIs (all imaging sessions) were expressed as one vector. From this, task-modulated ROIs were removed and Pearson’s correlation coefficient for α- and α’-responses was calculated. This value was compared against the correlation coefficient obtained from the full set i.e., change in correlation = coefficient (ROI removed) – coefficient (full set))/coefficient (full set). For removing random sets of ROIs, a random subset, comprising the same number of ROIs as the task-modulated set, was selected using the randperm function in Matlab. Then the change in correlation was calculated as above.

#### Linear decoder analysis

Matlab function fitcdiscr was used to fit a linear classifier. For each dataset (each imaging session), training data comprised the population of M/TC responses from 2/3 of the trials during the fine discrimination block. The other 1/3 of the trials was used as a test set to predict the label (S+ vs S- odour). % accuracy is the % of trials from the test set that matched the correct label.

#### Behavioural performance

Animal lick responses were analysed. For S+ and S- trials, correct responses were the presence and absence of licking during a window 1–2.5 s after odour onset, respectively. Incorrect responses were the absence and presence of lick responses for S+ and S- trials in the same window, respectively. The number of correct trials was expressed as the % of all trials.

## Additional files

Transparent reporting form

### Data availability

All data generated or analysed during this study are included in the manuscript and supporting files.
